# The Kidney Injury Induced by Short-Term PM_2.5_ Exposure and the Prophylactic Treatment of Essential Oils in BALB/c Mice

**DOI:** 10.1155/2018/9098627

**Published:** 2018-07-29

**Authors:** Yining Zhang, Qiujuan Li, Mengxiong Fang, Yanmin Ma, Na Liu, Xiaomei Yan, Jie Zhou, Fasheng Li

**Affiliations:** ^1^College of Medical Laboratory, Dalian Medical University, Dalian, Liaoning Province 116044, China; ^2^Department of Preventive Medicine Laboratory, Dalian Medical University, Dalian, Liaoning Province 116044, China; ^3^Clinical Laboratory, Dalian Municipal Friendship Hospital, Dalian, Liaoning Province 116001, China

## Abstract

PM_2.5_ is well known as a major environmental pollutant; it has been proved to be associated with kidney diseases. The kidney damage involves oxidative stress and/or inflammatory response. NOX4 is a major source of reactive oxygen species (ROS) generation in the kidney, and the excessive generation of ROS is recognized to be responsible for oxidative stress. To elucidate whether short-term PM_2.5_ exposure could induce kidney damage, we exposed BALB/c mice to PM_2.5_ intratracheally and measured the biomarkers of kidney injury (KIM-1, cystatin C), oxidative stress (MDA, SOD-1, and HO-1), and inflammatory response (NF-*κ*B, TNF-*α*). Acute kidney damage and excessive oxidative stress as well as transient inflammatory response were observed after PM_2.5_ installation. The overexpression of some components of the angiotensin system (RAS) after PM_2.5_ exposure illustrated that RAS may be involved in PM_2.5_-induced acute kidney injury. CEOs (compound essential oils) have been widely used because of their antioxidant and anti-inflammation properties. Treatment with CEOs substantially attenuated PM_2.5_-induced acute kidney injury. The suppression of RAS activation was significant and earlier than the decrease of oxidative stress and inflammatory response after CEOs treatment. We hypothesized that CEOs could attenuate the acute kidney injury by suppressing the RAS activation and subsequently inhibit the oxidative stress and inflammatory response.

## 1. Introduction

PM_2.5_ is the tiny particular with an aerodynamic diameter less than 2.5 *μ*m. PM_2.5_ comprises a complex mixture with several components such as metals (arsenic, lead), organic components (polycyclic aromatic hydrocarbons, organic carbon), and biological components (bacteria, fungal spores, and endotoxin). The particle is so tiny that can easily deposit in the alveoli even pass directly through the alveolar capillaries into the bloodstream, so it can lead to several of pulmonary and cardiovascular diseases associated with the increased risk of cancer. Earlier studies showed that PM_2.5_ exposure caused damage to the respiratory system and result in some acute and chronic respiratory diseases, even increasing the risk of lung cancer [1]. Nowadays, the exploration of PM_2.5_ is not only limited to the respiratory system. Recent studies demonstrated that PM_2.5_ can easily pass through the alveolar epithelial cells, enter into the circulatory system, and finally damage the cardiovascular system as well as the kidney [2, 3]. A previous study reported that long-term exposure to PM_10_ reduced the renal function [4]. Recent studies have informed that long-term PM_2.5_ exposure negatively affects renal function and increase renal function decline [5]. A retrospective study in China has shown that long-term exposure to PM_2.5_ is associated with an increased risk of membranous nephropathy [6]. Furthermore, some studies have demonstrated that long-term exposure to outdoor PM can increase the risk of kidney parenchyma cancer [7, 8]. Lately, Aztatzi-Aguilar et al. demonstrated that subchronic exposure to PM_2.5_ induced an acute kidney damage that involved an angiotensin and bradykinin system as well as antioxidant and immune imbalance [9].

The applications of acute kidney injury molecule markers have improved the diagnosis of acute renal damage. Kidney injury molecule-1 (KIM-1) has emerged as a promising biomarker of proximal tubular damage which significantly increased as early as 3 h of renal I/R injury [10–13]. So the early diagnosis of renal dysfunction can be provided by detecting KIM-1. Cystatin C, an early sensitive indicator for detecting renal injury, due to its small size, can be easily filtered by the glomerulus and then completely reabsorbed by the renal tubules. So cystatin C has been recognized as a promising marker of glomerular filtration rate (GFR), which is superior to the traditional indicator (serum creatinine) in sensitivity and timeliness and now used widely in clinics [11, 12]. The kidney is responsible for the elimination of 70% of the daily uric acid (UA) production; when renal injury occurs, the filtration of UA is reduced and the serum UA concentration is increased, so UA detected in serum may be a simple marker of the imminent onset of AKI [14, 15].

Oxidative stress (OS) refers to the imbalance between free radicals and antioxidants in vivo [16]. The increase of ROS generation can cause apoptosis and tissue damage and induce various diseases [17]. NADPH oxidase (NOX), including Nox1, Nox2, Nox3, Nox4, Nox5, Duox1, and Duox2 [18], is a family of membrane-associated multisubunit enzymes that represent a major source of creative oxygen species (ROS) in the kidney [19] NOX4 is a member of the NADPH family which is highly expressed in the kidney and upregulated in sustained oxidative stress response. NOX4 has been proposed to be a key actor as diverse as oxygen sensing in the kidney [16, 20]. NF-*κ*B is a transcription factor that regulated biological processes such as inflammation and apoptosis proliferation; activated NF-*κ*B plays a pivotal role in the control of several genes, including cytokines (TNF-*α*, IL-1*β*, and IL-6), chemokines, and adherence molecules (ICAM-1), and finally leads to organ injury [21, 22]. Oxidative stress, together with the inflammatory response, had been recognized as the underlying mechanism for the harmful effects as a consequence of PM_2.5_ exposure [9, 23].

The kidney is one of the metabolic organs in the human body which maintains body fluid balance and acid-base balance. The RAS (renin-angiotensin system) is an important body fluid-regulating system, maintains the basic function, and affects the physiological and pathological progress of the kidney [24]. Angiotensin II (Ang-II) is the major bioactive peptide of RAS, which is obtained from its precursor molecule, angiotensinogen (AGT). AGT is first converted by renin to produce a decapeptide, angiotensin I (Ang-I), which is then converted to Ang-II by removal of a COOH-terminal dipeptide by angiotensin-converting enzyme (ACE). Almost all the harmful effects of Ang-II are mediated by AT1 receptors (AT1R); AT1R drives renal and vascular inflammation during hypertension which is particularly abundant in the kidney [25]. It is known that Ang-II is a positive regulator which can induce oxidative stress by stimulating NADPH oxidase activity as well as inflammatory response [26].

Essential oils (EOs) are extracted from plants, flowers, leaves, stems, roots, or fruits by steam distillation, extrusion method, soaking method, or solvent extraction method which has been used in medicine, pharmaceuticals, perfumery, cosmetics, and many food applications. EOs have attracted more and more people's attention because of the properties of antioxidant, anti-inflammatory, and antimicrobial activities. Some components in compound essential oils also have the effects of adrenal support, relieved sedation, and enhanced immunity [27]. Generally, EOs are comprised of two or more essential oils that display better effects than simple component due to synergic interactions. Recent studies have confirmed that mint, eucalyptus, spruce, and frankincense EOs possess strong anti-inflammatory and antioxidant activities [28–32]. In this study, those four EOs were mixed together and the effect of CEOs on acute kidney injury induced by PM_2.5_ exposure was evaluated.

The aim of our study was to investigate whether short-term PM_2.5_ exposure could lead to kidney injury and to explore the underlying mechanism of the response. We measured the potency of CEO-associated PM_2.5_ exposure through modulating RAS-induced oxidative stress and inflammatory response.

## 2. Materials and Methods

### 2.1. Animals

6–8-week-old male BALB/c mice were purchased from Changsheng Biotechnology Co., Ltd. (Shenyang, China). Animals were kept in a conditioned room (24 ± 1°C) with a 12/12 h light/dark cycle with free access to water and food. All animals were fully anesthetized before the experiment to reduce the pain of animals. All animal procedures were executed in strict conformation with the local institute of the Animal Care and Use Committee of Dalian Medical University.

### 2.2. PM_2.5_ Suspension

Particulate matters were collected in Langfang (Hebei, China), from December 2013 to March 2014 by a PM_2.5_ high volume air sampler (Thermon Anderson, USA) using ultrafine quartz fiber filters (General Electric, USA). The filter with PM_2.5_ was cut and put into the sterile distilled water and subsequently administrated by ultrasonic sonication for 2 hours. The PM_2.5_ suspension separated from the filter was then vacuum freeze-dried, weighed, and stored at −20°C [33, 34]. PM_2.5_ suspension with 10 mg/ml concentration in saline was prepared for exposure experiment. Previous study had measured the elements of the suspension which included organic carbon elemental carbon, Zn, Pb, Cu, NH_4_^+^, NO_3_^−^_,_ and SO_4_ [2–35].

CEOs mint, eucalyptus, spruce, frankincense, and rose hip EOs were supplied by Absolute Aromas Ltd. (4 Riverway, Alton, GU34 2QL, England). The purity of all above was 100%. According to aromatherapy, CEOs were compounded with mint, eucalyptus, spruce, and frankincense including eucalyptol, a-pinene, and menthol [35].

### 2.3. Chemicals and Antibodies

A lipid peroxidation MDA assay kit (S0131) and a hydrogen peroxide assay kit (s0038) as well as a total glutathione peroxidase assay kit (s0058) were purchased from Beyotime Biotechnology (Shanghai, China). BCA protein assay kit (KGP902) and ECL detection kit were purchased from KeyGENE (Nanjing, China). PrimeScript™ RT reagent kit with gDNA Eraser (Perfect Real Time) (code number RR047A) and SYBR® Premix Ex Taq™ II (Tli RNaseH Plus) (code number RR820A) were purchased from TaKaRa. *β*-Actin monoclonal antibody was purchased from Zhongshan Jinqiao (Beijing, China). Rabbit polyclonal NOX4 antibody (14347-1-AP) and rabbit polyclonal AGTR1 antibody (25343-1-AP) were purchased from Proteintech (Wuhan, China). NF-*κ*B-p65 antibody and NF-*κ*B-pp65 (ser536) antibody were obtained from Wanleibio (Shenyang, China). Rabbit polyclonal TNF-*α* antibody was purchased from Bioworld (Nanjing, China). Rabbit polyclonal ACE antibody was purchased from ABclonal (Wuhan, China).

### 2.4. Experiment Design

Experimental procedures were divided into two parts. In the first part, 48 BALB/c mice were divided into two groups randomly (*n* = 24): control group—intratracheal instillation of 50 *μ*l sterile saline on day 0 and day 2—and PM_2.5_ group—intratracheal instillation of 50 *μ*l aqueous PM_2.5_ suspensions (0.5 mg PM_2.5_ in sterile saline) on day 0 and day 2. About 8 mice were selected randomly each time in both the control group and the PM_2.5_ group and then sacrificed on days 3, 7, and 14 after intratracheal instillation.

In the second part, 48 BALB/c mice were divided into two groups randomly (*n* = 24), as follows: PM_2.5_ + saline group—static inhalation of 200 *μ*l of sterile saline the day before PM_2.5_ exposure (intratracheal instillation of 50 *μ*l aqueous suspensions of 0.5 mg PM_2.5_ in sterile saline at day 0 and day 2)—and PM_2.5_ + CEO group—constant daily static inhalation of 200 *μ*l of CEOs for 30 min per day until killed on the base of PM_2.5_ exposure which was described previously. About 8 mice were selected randomly each time in both groups and then sacrificed on days 3, 7, and 14 after intratracheal instillation.

### 2.5. Tissue and Serum Collection

After 3, 7, and 14 days' treatment of intratracheal instillation, mice were sacrificed. The kidney and blood were immediately removed. A longitudinal cross-section of each kidney was fixed in 4% paraformaldehyde for HE (hematoxylin-eosin) staining; the remaining portion of the kidney was frozen quickly with liquid nitrogen and stored at −80°C subsequently for latter gene expression and protein abundance. Blood samples were centrifuged at 4°C, and the supernatants of serum were stored at −80°C for the measurement of cystatin C and UA.

### 2.6. Pathological Examination

Mouse kidney tissues were fixed in 4% paraformaldehyde, dehydrated, embedded in paraffin and cut into sections of 6 *μ*m, and finally stained with hematoxylin and eosin (H&E). The histopathological lesions and changes were observed under the inverted microscope. Six random noncoincident microscopic fields were assessed per animal.

### 2.7. The Measurement of Oxidative Stress

The measurement of MDA was based on the method of the color reaction between thiobarbituric acid (TBA) and the lipoperoxidation product malondialdehyde, which produced a red-colored complex that can be measured by spectrophotometry at 532 nm. The results were expressed as nanomole per milligram of protein (nmol/mg protein). The level of hydrogen peroxide and glutathione peroxidase activity were determined according to the instructions of the kit. The results were expressed as micromole per milligram of protein (*μ*mol/mg protein) and unit per milligram of protein (U/mg protein).

### 2.8. Western Blot Analysis

Kidney tissues about 50 mg were cut into small pieces. The supernatant was extracted to determine the protein concentration by the BCA protein assay kit. Equal amounts of protein (40 *μ*g) were run on a 12% SDS-polyacrylamide electrophoresis gel and transferred onto a 4.5 mm PDF membrane. Membranes were blocked for 2 h in 10% nonfat milk at 37°C in a shaker and then incubated with the primary antibodies including AGTR1 (1 : 2000), ACE (1 : 1000), NOX4 (1 : 500), NF-*κ*B p65 (1 : 1000), NF-*κ*B pp65 (1 : 1000), and TNF-*α* (1 : 1000) overnight at 4°C and incubated the corresponding secondary antibodies for 2 h at room temperature. Proteins were detected with an enhanced chemiluminescence (ECL) detection kit. The bands were finally quantified by ImageJ software. All protein levels in our study were achieved by Western blot.

### 2.9. Analysis of Gene Expression

The total kidney RNA was extracted using TRIzol reagent (Invitrogen, Carlsbad, CA, USA) according to the manufacturer's instruction; RNA concentrations were determined by a microplate reader. 1 *μ*g of RNA was separated by gDNA eraser in 20 *μ*l. Total cDNA was synthesized with the PrimeScript RT reagent kit with gDNA eraser and using a T100 Thermal Cycler (Applied BIO-RAD, Hercules City, CA, USA). Real-time (RT) polymerase chain reaction (PCR) was performed using an SYBR Premix Ex Taq II (Tli RNaseH Plus) kit and TP800 Thermal Cycler Dice (Applied Real-Time System). All experiments were performed independently at least three times, and each time, we do two parallel holes to control the artificial error. All of the gene expressions in our study assays were done this way.

### 2.10. Statistical Analysis

Data were expressed as the mean ± standard errors (mean ± SEM). All statistical analysis was performed using GraphPad Prism 5.0 for Windows (GraphPad Software, San Diego, CA). Statistical significance was assessed by Student's *t*-test. A *P* value less than or equal to 0.05 was considered statistically significant.

## 3. Results

### 3.1. Kidney Damage Induced by PM_2.5_ Exposure

**Figure 1:**
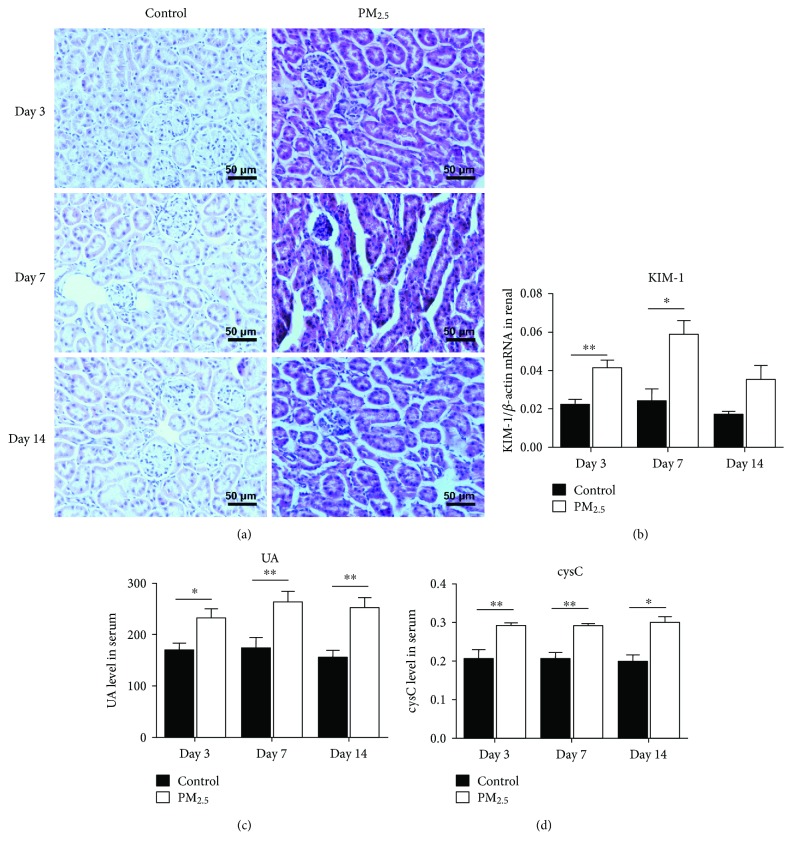
Mouse kidney injury after administration by PM_2.5_ or saline (bar = 50 *μ*m). Samples were stained using H&E, no significant pathological change in the PM_2.5_ group compared with the control group (a). KIM-1 mRNA was assayed by RT-PCR, serum UA and cystatin C were assessed using chemiluminescence. KIM-1 mRNA expression of kidney tissues (b) and serum UA (c) and serum cystatin C (d) increased after exposure (*n* = 6, ^∗∗^*P* < 0.01 and ^∗^*P* < 0.05 versus the control group).

The histopathological changes of kidney tissues were observed by H&E staining. A previous study demonstrated that short-term PM_2.5_ exposure induced acute airway injury which can be reflected in H&E staining [35]. But observing the results by using an optical microscope, we found that tissue edema occurred in the individual renal samples. And there was no significant difference between the control group and the PM_2.5_ group (Figure 1(a)). However, the changes in serum kidney injury indexes and tissue KIM-1 gene expression were apparent. Both serum cystatin C (Figure 1(c)) and UA (Figure 1(d)) were remarkably increased from the beginning of PM_2.5_ exposure. The mRNA expression of KIM-1 was evaluated by real-time (RT) polymerase chain reaction (PCR). The gene expression of KIM-1 was elevated on day 3 and day 7 (Figure 1(b)).

### 3.2. PM_2.5_ Exposure Induced Oxidative Stress through Overexpression of NOX4 and Subsequently Activated Inflammatory Response

**Figure 2:**
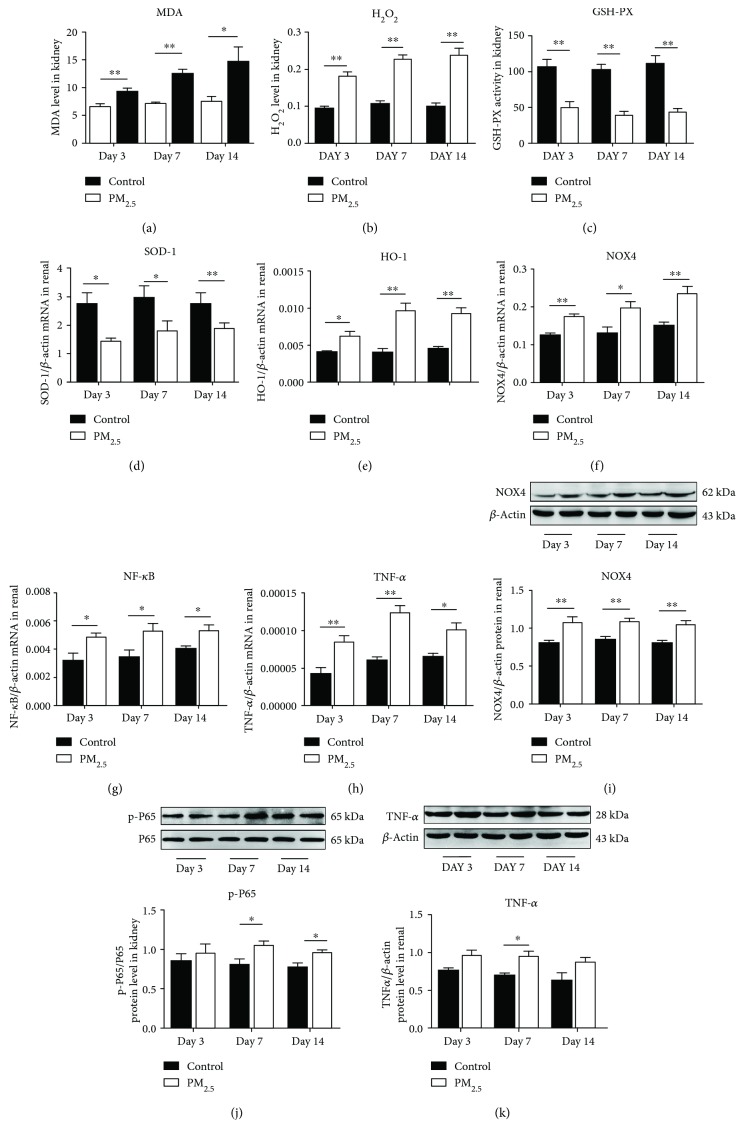
PM_2.5_ induced oxidative stress and inflammatory response. PM_2.5_ increased MDA (a) and H_2_O_2_ (b) level (*n* = 6). The mRNA expression of HO-1 (e), NOX-4 (f), NF-*κ*B (g), and TNF-*α* (h) was promoted and SOD-1 (d) as well as GSH-PX (c) was suppressed by PM_2.5_ (*n* = 6)_._ The protein generation of NOX4 (i), NF-*Κ*b (j), and TNF-*α* (k) was in accordance with their gene levels (*n* = 4). ^∗∗^*P* < 0.01 and ^∗^*P* < 0.05 versus the control group.

As an indicator of oxidative stress, the MDA of kidney tissues in the PM_2.5_ group was gradually increased compared with that in the control group since day 3 (Figure 2(a)). H_2_O_2_ as the most direct product of NOX4 should be determined; the content of H_2_O_2_ increased significantly in the kidney since the early stage of PM_2.5_ exposure (Figure 2(b)). Glutathione peroxidase (GSH-PX) is an important peroxidase in the body and promotes the decomposition of H_2_O_2_ to weaken the oxidative stress reaction. We found that the content of H_2_O_2_ scavenger GSH-PX was significantly reduced in mouse kidney after PM_2.5_ exposure (Figure 2(c)). We also assessed the important antioxidant enzyme; the gene expression of SOD-1 showed a considerable decrease at all time points in the PM_2.5_ group (Figure 2(d)). Interestingly, HO-1 is another protective enzyme of oxidative injury, in which mRNA expression increased all the time after PM_2.5_ exposure (Figure 2(e)). Studies have shown that NOX4 is a member of the NADPH oxidase family and recognized as the main source of kidney ROS generation. Firstly, gene expression of NOX4 was measured and it indicated that the mRNA of NOX4 presented an obvious increase since day 3 after PM_2.5_ exposure (Figure 2(f)). The protein level of NOX4 (Figure 2(i)) was measured subsequently. The consensus between protein and gene expression confirmed our speculation that PM_2.5_ exposure might induce oxidative stress through NOX4 excessive generation.

The NF-*κ*B and TNF-*α* were measured to evaluate the effect on inflammatory response induced by PM_2.5_ exposure. As shown in Figure 2(g), the mRNA expression of NF-*κ*B increased at all time points, while the protein level of pp65 only slightly increased on day 7 (Figure 2(j)). The mRNA expression of TNF-*α* (Figure 2(h)) increased on day 3 and day 7, and the protein level elevated on day7 (Figure 2(k)).

### 3.3. PM_2.5_ Exposure Induced Oxidative Stress and Inflammatory through RAS Activation

**Figure 3:**
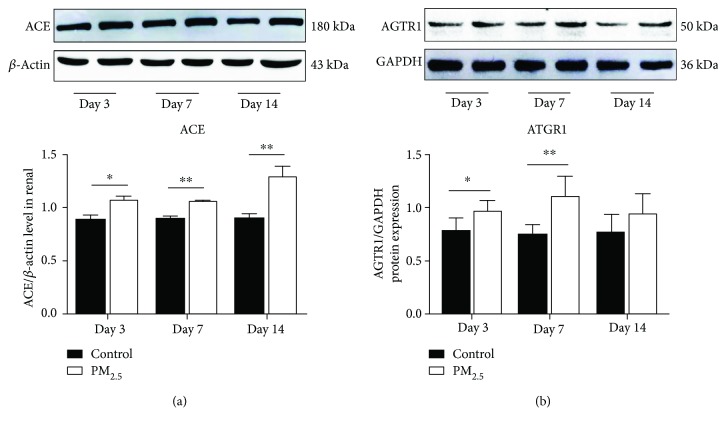
PM_2.5_ activated the RAS. An augment in the angiotensin-converter enzyme (ACE) (a) and angiotensin-receptor type-I (AT1R) (b) after PM_2.5_ exposure (*n* = 4, ^∗∗^*P* < 0.01 and ^∗^*P* < 0.05 versus the control group).

Ang-II is one of the most important stimuli to promote oxidative stress. It was recognized as a proinflammatory modulator. As shown in Figure 3, marked increase of ACE protein level was found in the PM_2.5_ group compared with the control group (Figure 3(a)). It was shown that the protein expression of AGTR1 increased significantly on day 3 and day 7 (Figure 3(b)). Signs of RAS activation, increased angiotensin-converting enzyme activity, and enhanced AT1R protein expression all demonstrated that PM_2.5_ exposure activated the RAS system in mouse renal.

### 3.4. Inhibitory Effects of CEOs on PM_2.5_ Induced Acute Kidney Injury

**Figure 4:**
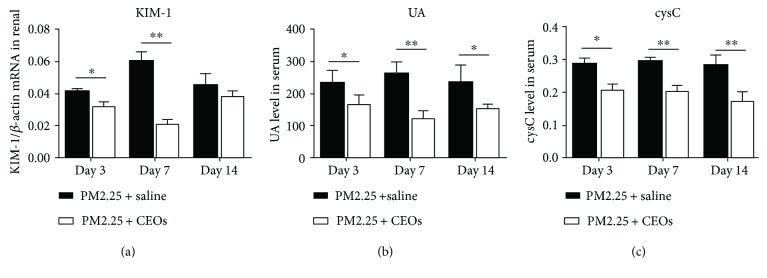
CEOs alleviated acute kidney injury. KIM-1 mRNA expression in kidney tissues (a), serum UA (b), and serum cystatin C (c) decreased after administration of CEOs (*n* = 6, ^∗∗^*P* < 0.01 and ^∗^*P* < 0.05 versus the PM_2.5_ + saline group).

Previous studies had demonstrated that EOs have antioxidant, anti-inflammatory, antifungal and antinociceptive properties. To investigate the functions of the antioxidant effect of CEOs in the PM_2.5_-exposed model, static exposure was used to induce mice to inhale CEOs or an equal volume of saline after PM_2.5_ exposure. Kidney tissues and serum were collected when mice were sacrificed on day 3, day 7, and day 14. There was no significant change in the PM_2.5_ + CEO group compared with the PM_2.5_ + saline group by observing H&E staining. When using chemiluminescence to detect serum kidney injury index, both serum cystatin C (Figure 4(c)) and UA (Figure 4(b)) were downregulated in the PM_2.5_ + CEO group at all three time points compared with those in the PM_2.5_ + saline group. The mRNA of KIM-1 also decreased on day 3 and day 7 especially on day 7 (Figure 4(a)). These results demonstrated that CEOs reduced PM_2.5_-induced acute kidney injury.

### 3.5. Inhibitory Effects of CEOs on PM_2.5_ Induced Oxidative Stress and Inflammatory Response

**Figure 5:**
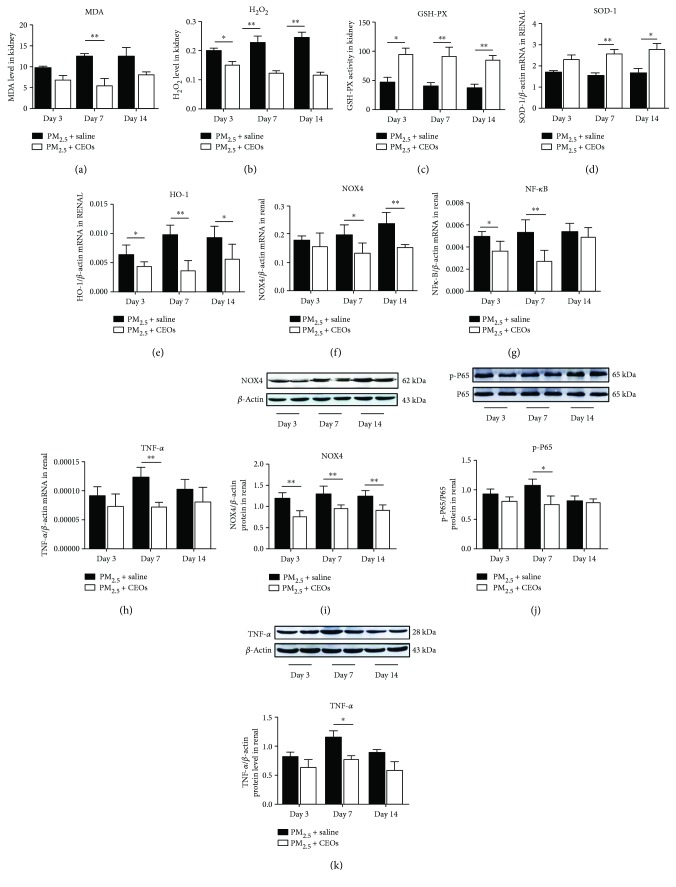
CEOs decreased oxidative stress and inflammatory response. MDA level (a), H_2_O_2_ level (b), and the mRNA expression of HO-1 (e), NOX-4 (f), NF-*κ*B (g), and TNF-*α* (h) were suppressed; SOD-1 mRNA expression (d) and the level of GSH-PX (c) were increased (*n* = 6). The protein level of NOX-4 (i), NF-*κ*B (j), and TNF-*α* (k) decreased after the treatment of CEOs (*n* = 4) (^∗∗^*P* < 0.01 and ^∗^*P* < 0.05 versus the PM_2.5_ + saline group).

To access the activity of CEOs against oxidative stress, the ROS and some antioxidants were measured. Figures 5(a) and 5(b) show that the kidney MDA and H_2_O_2_ in the PM_2.5_ + CEO group were significantly downregulated since day 7. The level of GSH-PX increased sharply as soon as the treatment of CEOs (Figure 5(c)). The gene expression of SOD-1 (Figure 5(d)) recovered since day 7, but the mRNA level of HO-1 (Figure 5(e)) decreased at all three time points. To further understand the mechanism underlying the CEO-induced reduction of ROS generation, the inhibitory effect of CEOs on NOX4 was followed. Compared with the PM_2.5_ + saline group, there was an obvious decrease of both gene (Figure 5(f)) and protein expressions (Figure 5(i)) since day 7. These results well demonstrated that CEOs could inhibit oxidative stress mediated by NOX4.

In order to prove whether CEOs could reduce the inflammatory response, we measured the gene expression of NF-*κ*B and TNF-*α* as well as the protein level of NF-*κ*B pp65 and TNF-*α*. As the pivotal transcription factor of various inflammatory reaction, the mRNA expression of NF-*κ*B decreased on day 3 and day 7 (Figure 5(g)). However, the protein level of NF-*κ*B pp65 a form of phosphorylation which represents the activation of NF-*κ*B only decreased on day 7 (Figure 5(j)). Both gene (Figure 5(h)) and protein expressions (Figure 5(k)) of TNF-*α* in the PM_2.5_ + CEO group were lower on day 7 than those in the PM_2.5_ + saline group. These results indicated that CEOs could suppress the transient inflammatory response induced by short-term PM_2.5_ exposure.

### 3.6. CEOs Could Suppress Oxidative Stress and Subsequent Inflammatory Response through Inhibiting the RAS System Activation

**Figure 6:**
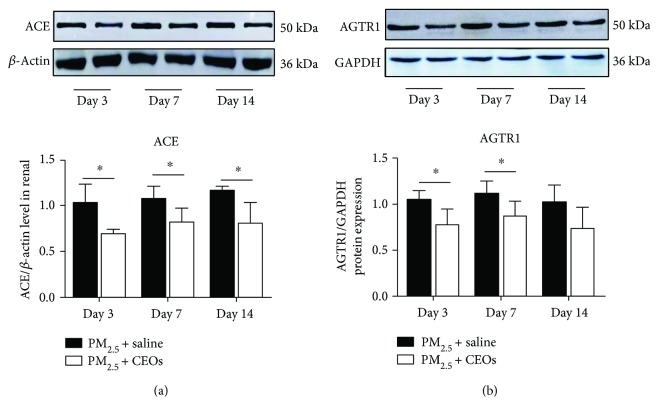
CEOs suppressed the RAS. ACE (a) and AGTR1 (b) were downregulated in the early stage (*n* = 4, ^∗∗^*P* < 0.01 and ^∗^*P* < 0.05 versus the PM_2.5_ + saline group).

Changes in protein levels were determined by Western blot assay. ACE in the PM_2.5_ + CEO group decreased significantly at three time points (Figure 6(a)). Treatment with CEOs and AGTR1 declined on day 3 and day 7 (Figure 6(b)). The results indicated that the CEOs' inhibitory effects on RAS were a priority to the inhibitory effects on oxidative stress and inflammatory response. So the CEOs could restrain PM_2.5_-induced RAS system activation and reduce the subsequent reactions such as oxidative stress and inflammatory response.

## 4. Discussion

PM_2.5_ has become a major air pollutant which is closely related to the diseases of the respiratory system and cardiovascular system [36]. In recent years, more and more researchers have shifted their attention from the lung and heart to the circulatory system such as the kidney [4–7, 9, 37, 38]. But acute kidney injury to acute PM_2.5_ exposure has not been revealed clearly. A previous study had shown that short-term PM_2.5_ exposure could induce the airway inflammation [35]. In our study, we used BALB/c mice to investigate whether short-term PM_2.5_ exposure can induce kidney damage through activating oxidative stress and subsequent inflammatory response. To evaluate the potential mechanism, the changes in the renin-angiotensin system after PM_2.5_ exposure were investigated. CEOs suppressed PM_2.5_-induced acute kidney injury by inhibiting oxidative stress and inflammation which all might be activated by RAS. It indicated that the effect of the essential oils was achieved by counteracting RAS activation, inhibited downstream oxidative stress and inflammation, and then achieved a prophylactic treatment effect on kidney injury caused by PM_2.5_.

Considering that KIM-1 and cystatin C can be detected as the acute kidney damage markers represent renal tubular injury and glomerular injury [13, 39], we measured the two markers and found both of them rise in the PM_2.5_ exposure group. On the contrary, the results of H&E staining had no significant difference between the PM_2.5_ group and the control group. These results illustrated that short-term PM_2.5_ exposure damaged the kidney at the molecular level but has not caused kidney visible injury yet.

Renal damage caused by PM_2.5_ can be interpreted as the result of oxidative stress and inflammatory response [40–42]. MDA and H_2_O_2_ were considered the direct markers for oxidative stress [43], and the level of GSH-PX and gene expression of HO-1and SOD-1 are regarded as the antioxidant stress indexes. The increase of MDA and H_2_O_2_, as well as the decrease of GSH-PX and SOD-1, confirmed the activation of oxidative stress after PM_2.5_ exposure. The results showed a high level of HO-1 after the short-time exposure, which could be due to the activation of Nrf2/ARE [44, 45]. It was hypothesized that in short-term PM_2.5_ exposure, due to the increase of HO-1, part of the inflammatory response was suppressed, resulting in a transient increase in inflammatory response. Because of the close relationship between NOX4 and oxidative stress-induced kidney diseases, we measured the mRNA and protein of NOX4 [46–48]. H_2_O_2_ was the main product of NOX4. Glutathione peroxidase (GSH-PX) is an important peroxidase in the body and promotes the decomposition of H_2_O_2_ to weaken the oxidative stress reaction [49, 50]. The increase of H_2_O_2_ and the decrease of H_2_O_2_ scavenger GSH-PX also indicated the activation of upstream NOX4. That is to say, PM_2.5_ exposure could lead to the oxidative stress and is probably achieved by increasing the overexpression of NOX4 in the kidney.

Previous studies reported that the phosphorylation of p65 at Ser-536 within the transactivation domain was mediated by a number of protein kinases and stimulated transcriptional activity [51–53]. The gene expression of NF-*κ*B and TNF-*α* in the PM_2.5_ exposure group is higher than that in the control group, but the protein level of pp65 and TNF-*α* was only increased on day 7. These results illustrated that PM_2.5_-induced inflammatory response in the kidney may be a transient elevation.

Ang-II is the main stimulus-induced oxidative stress which, via an AT1 receptor, especially modulates the production of ROS through NADPH oxidase activation [54–57]. The latest study showed that rats after intratracheal instillation with PM_2.5_ displayed increased circulating level of Ang-II, the major bioactive peptide in the renin-angiotensin system (RAS), which resulted from the elevation of Ang-II production in the vascular endothelium [58]. In the diabetic kidney injury model, oxidative stress induced by the overexpression of Ang-II is considered to be the major pathway of injury [59–61]. Besides, Ang-II is involved in the activation of inflammatory response by activating NF-*κ*B [62–64]. In Ang-II-infused animals, NF-*κ*B activation increased and the expression of TNF-*α*, IL-6, and MCP-1 is augmented [62]. In mesangial cells, Ang-II activated NF-*κ*B which has been identified as an important protein that initiates the transcription [64]. Hence, the blockade of Ang-II has been considered a major therapeutic strategy for hypertension nephropathy. To verify the effect on the kidney after PM_2.5_ exposure, we tested the changes of the major renal physiological regulatory system. So we measured ACE and angiotensin receptor type-1 (AT1R) in kidney tissues after PM_2.5_ exposure. In our experimental model, PM_2.5_ exposure increased kidney protein level of AT1R and ACE; the rapid rise of these cytokines in the early time point suggested the RAS is likely to be an important upstream pathway for the initiation of oxidative stress and inflammatory response.

Previous studies have illustrated some herbal medicines can inhibit oxidative stress and inflammatory response by inhibiting the RAS system [65, 66]. In our study, mice were administrated CEOs or saline via static inhalation after PM_2.5_ exposure. We found CEOs significantly restrained PM_2.5_-induced kidney oxidative stress and inflammatory response to alleviate the renal damage. These findings reminded us the importance of CEOs in suppressing oxidative stress and inflammation induced by PM_2.5_ exposure. To investigate the underlying mechanism, we assessed the RAS in the kidney. The decreased protein level of ACE and AT1R implied that CEOs may inhibit PM_2.5_-induced RAS activation. At the same time, we observed the changes of these indicators at three time points and found that the CEOs' inhibitory effect on oxidative stress and inflammatory response occurred at the later stage of our study; however, the suppression of CEOs on RAS was earlier than oxidative stress and inflammatory response. We hypothesized that RAS may be the upstream pathway of oxidative stress and inflammatory response. But the early suppression of these kidney injury indicators implied us that in addition to the mechanism we have studied, CEOs are likely to inhibit kidney injury through other pathways.

## 5. Conclusions

It was found that short-term exposure to PM_2.5_ induced acute kidney damage associated with oxidative stress and inflammatory response. The activation of RAS might be the pivotal upstream of oxidative stress and inflammatory response induced by PM_2.5_ exposure. In addition, CEOs mixed with mint, eucalyptus, spruce, and frankincense reduced PM_2.5_-induced acute kidney injury; the antioxidant and anti-inflammation function of CEOs was well proved. The earlier suppression of RAS indicated that CEOs might alleviate kidney injury through the RAS. Advanced interfere treatment could prevent further deterioration of renal damage to achieve a purpose of prophylactic treatment. These findings might provide potential mechanisms and a novel therapy for PM_2.5_-related kidney diseases.

## Data Availability

The data used to support the findings of this study are available from the corresponding author upon request.
